# Supplementary material for: Functional conservation and coherence of HIV-1 subtype A Vpu alleles
Source: Sci Rep. 2017 Mar 7;7:87. doi: 10.1038/s41598-017-00222-8 (PMC5428049; doi:10.1038/s41598-017-00222-8)
Supplement: Supplementary file 1 — Supplementary file [file 41598_2017_222_MOESM1_ESM.pdf]

Functional conservation and coherence of HIV-1 subtype A Vpu alleles

Bizhan Romani[1,2], Amirarsalan Kavyanifard[3], and Elham Allahbakhshi[1*]

[1]Cellular and Molecular Research Center (CMRC), Faculty of Medicine, Ahvaz Jundishapur University of Medical Sciences (AJUMS), Ahvaz, 61357-15794, Iran

[2]Department of Biology, Faculty of Science, University of Isfahan, Isfahan, 81746-73441, Iran

[3]Department of Biology, Payam Noor University, Tehran, 1395-4697, Iran

Running title: HIV-1 subtype A Vpu

***Corresponding author:** Elham Allahbakhshi

Tel: +98 916 113 7679; Fax: +98 613 333 6380; E-mail: abakhshi_e@ajums.ac.ir

Table S1. Vpu mutations and prediction of their impact on protein function

Mutation(s)	SIFT score	Predicted tolerated amino acids*
L2S, L2T, L2P	L=0.35, S=0.83, T=0.1, P=0.2	h c m f y r q I d e g P k n v L a S T
P3S	P=1.00, S=0.89	g t a S P
L4F	L=1.00, F=0.13	F L
D5H, D5Q, D5E, D5H	D=0.66, H=0.19, Q=0.28, E=1.00, H=0.19	v l r t p g s n H a k Q D E
I6T, I6V	I=1.00, T=0.27, V=0.39	a l T V I
W7C, W7I	W=1.00, C=0.14, I=0.26	m t a C v y f l I W
A8T	A=1.00, T=0.29	s T A
V10T, V10I	V=1.00, T=0.28, I=0.38	a l T I V
G11A	G=1.00, A=0.18	A G
V13I	V=1.00, I=0.28	I V
I20V	I=1.00, V=0.20	V I
V21G, V21I	V=1.00, G=0.09, I=0.49	G l I V
V22M	V=1.00, M=0.12	l i M V
E29Q	E=1.00, Q=0.74	d Q E
I30Y, I30F	I=1.00, Y=0.31, F=0.35	v l Y F I
K31R	K=1.00, R=0.17	R K
R32G, R32D, R32K, R32E	R=0.63, G=0.37, D=0.68, K=0.87, E=1.00	m h i v p l G n t q s a R D K E
L33M, L33F,L33I	L=1.00, M=0.17, F=0.17, I=0.34	v M F I L
L34K	L=1.00, K=0.15	r m v i K L
R35K	R=1.00, K=0.49	K R
K38R	K=1.00, R=0.17	R K
D40N, D40E	D=1.00, N=0.15, E=0.19	N E D
R41S	R=1.00, S=0.13	K S R
I42L	I=1.00, L=0.18	v L I
I43L	I=1.00, L=0.18	v L I
K44S, K44Q, K44N	K=1.00, S=0.28, Q=0.37, N=0.42	p l g t a d r e S Q N K
S47R	S=1.00, R=0.58	i h l v p d e q n g a t k R S
D58E	D=1.00, E=0.16	E D
D62E	D=1.00, E=0.74	E D
E63K, E63D	E=1.00, K=0.13, D=0.17	K D E
A65S	A=1.00, S=0.13	S A
E69G	E=1.00, G=0.14	k q a d G E
N72H, N72Y, N72D	N=1.00, H=0.19, Y=0.25, D=0.46	l f p r q t a k e H s Y g D N
Y73H	Y=1.00, H=0.12	H Y
D74A	D=1.00, A=0.19	s n g e A D
L75I	L=1.00, I=0.13	I L
G76W	G=1.00, W=0.22	m i h c q f y v r l e t k W p n d s a G
D77N	D=1.00, N=0.16	N D
N79D	N=1.00, D=0.15	D N
N80D	N=1.00, D=0.15	D N

*Amino acid substitutions that occurred in Vpu alleles are shown in capital.

Table S2. Effect of HIV-1 Vpu alleles with confidence intervals of 95% (numbers in brackets). The two-tailed *p* values were calculated by comparison with the respective mock samples.

Alleles	CD4		SNAT1		HLA-C		Tetherin		Virus release		NF-κB	
	Expression, %	*p*	Expression, %	*p*	Expression, %	*p*	Expression, %	*p*	Fold change	*p*	Activity, %	*p*
**S52,56N**	98.0 (85.1, 110.9)	0.7764	96.1 (91.7, 100.5)	0.1542	96.1 (85.4, 106.6)	0.5050	64.0 (53.9, 74.1)	0.0022	5.9 (4.9, 6.9)	0.0015	83.6 (73.4, 95.8)	0.0597
**NL4.3**	32.1 (26.7, 37.5)	$p<0.0001$	23.2 (19.6, 26.8)	$p<0.0001$	97.3 (87.9, 106.7)	0.6032	29.3 (23.3, 35.3)	$p<0.0001$	11.3 (8.6, 14.0)	0.0008	11.4 (9.9, 12.9)	$p<0.0001$
**JU1**	29.6 (25.6, 33.7)	$p<0.0001$	14.7 (12.0, 17.4)	$p<0.0001$	35.1 (30.5, 39.7)	$p<0.0001$	22.3 (17.2, 27.4)	$p<0.0001$	12.1 (9.3, 14.9)	0.0015	3.2 (2.6, 3.8)	$p<0.0001$
**JU2**	28.1 (23.5, 32.7)	$p<0.0001$	13.3 (10.1, 16.5)	$p<0.0001$	12.3 (9.9, 14.7)	$p<0.0001$	31.8 (24.4, 39.2)	$p<0.0001$	10.4 (7.8, 13.0)	0.0021	3.9 (3.3, 4.5)	$p<0.0001$
**JU3**	25.0 (20.6, 29.4)	$p<0.0001$	16.5 (13.2, 19.8)	$p<0.0001$	16.5 (13.9, 19.1)	$p<0.0001$	33.1 (27.3, 38.9)	$p<0.0001$	14.2 (10.7, 17.7)	0.0018	4.8 (4.1, 5.5)	$p<0.0001$
**JU4**	28.4 (23.9, 32.9)	$p<0.0001$	11.5 (9.4, 13.7)	$p<0.0001$	98.6 (92.0, 105.2)	0.6974	38.7 (31.0, 46.4)	$p<0.0001$	10.6 (8.6, 12.6)	0.0008	2.5 (2.1, 3.0)	$p<0.0001$
**JU5**	28.7 (24.0, 33.5)	$p<0.0001$	16.4 (12.9, 19.9)	$p<0.0001$	53.2 (47.4, 59.0)	$p<0.0001$	19.9 (14.8, 25.0)	$p<0.0001$	14.1 (11.2, 17.0	0.0009	5.3 (4.7, 5.9)	$p<0.0001$
**JU6**	26.0 (21.7, 30.3)	$p<0.0001$	13.0 (9.9, 16.1)	$p<0.0001$	25 (21.4, 28.6)	$p<0.0001$	27.0 (21.6, 32.4)	$p<0.0001$	13.8 (11.7, 16.0)	0.0003	3.6 (3.2, 4.1)	$p<0.0001$
**JU7**	24.7 (20.3, 29.1)	$p<0.0001$	15.3 (12.9, 17.7)	$p<0.0001$	33.2 (29.4, 37.1)	$p<0.0001$	21.6 (15.5, 27.7)	$p<0.0001$	15.5 (12.2, 18.8)	0.0010	4.5 (3.9, 5.1)	$p<0.0001$
**JU8**	25.7 (22.6, 28.8)	$p<0.0001$	17.4 (13.9, 20.9)	$p<0.0001$	64.7 (57.0, 72.4)	0.0008	24.5 (18.1, 31.0)	$p<0.0001$	14.2 (10.6, 17.8)	0.0020	1.9 (1.6, 2.2)	$p<0.0001$
**JU9**	22.4 (19.7, 25.1)	$p<0.0001$	13.8 (11.1, 16.5)	$p<0.0001$	32.5 (28.3, 36.7)	$p<0.0001$	36.2 (29.9, 42.5)	$p<0.0001$	11.2 (8.7, 13.7)	0.0013	4.2 (3.8, 4.7)	$p<0.0001$
**JU10**	28.0 (23.7, 32.3)	$p<0.0001$	15.9 (12.8, 19.0)	$p<0.0001$	20.9 (18.2, 23.6)	$p<0.0001$	22.8 (18.3, 27.3)	$p<0.0001$	14.4 (11.3, 17.5)	0.0010	3.7 (3.3, 4.2)	$p<0.0001$

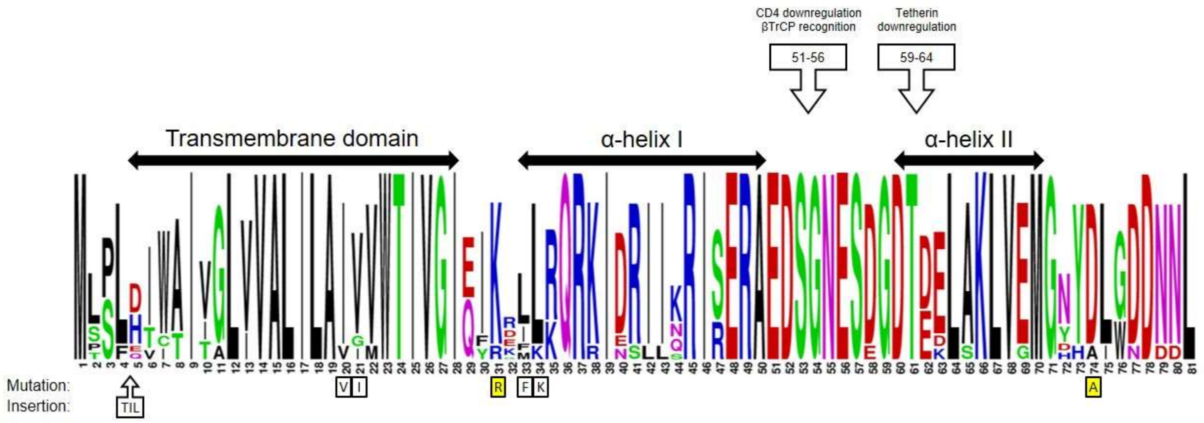

Figure S1. Consensus sequence of the clinical subtype A Vpu alleles.

Vpu domains and the known functional motifs are indicated above the consensus sequence. Amino acid mutations and insertions associated with inability for HLA-C downregulation are indicated under the consensus sequence. Mutations shared by the defective subtype A Vpu (JU4) and NL4.3 are highlighted in yellow.
